# Mental health symptoms: I wish they had known

**DOI:** 10.1038/s41537-022-00267-3

**Published:** 2022-07-26

**Authors:** Jason Jepson

**Affiliations:** Students With Psychosis, 3020 Hammock Street unit 1225, Myrtle Beach, SC 29577 USA

**Keywords:** Diseases, Schizophrenia

Things were not going well for me. I tried to find my way by going to college, working, training to be a firefighter—but nothing fit. And then—9/11 happened and my desire to do something for my wounded country emerged. The best way for me to respond was to join the Army. That direction would be a good fit. Little did I know that I was also in the early stages of a mental health journey.

So, I headed off to basic training; it was a grueling yet satisfying experience. The structure and comradery were a welcomed addition to my life. After graduation from basic and advanced training, I felt a deep sense of satisfaction and pride. The other privates in my platoon were like family. Unlike the National Guard privates who had trained with us, the soldiers in the “real army” were “shipped” off to their first duty stations. My first assignment resulted in a one-way ticket to southern California and the Mojave Desert. Leaving my graduation at Fort Knox, I became emotional as my parents drove me to the Louisville Airport. It hit me that I would never see the guys who had become like family. We did a U turn and returned to the post so I could say “goodbye” and wish all of my “buddies” all the best. We were all headed towards an uncertain future.

The Mojave Desert was a different world. I was a whole continent away from my home in Richmond, Virginia. Where I had once looked out on the green hills, I now looked out on the barren desert. Making friends in this new place seemed more difficult for me than at anytime in my life. I felt very lonely, and the remoteness of Fort Irwin created in me a feeling of isolation that I had never experienced before. I grew lonelier every day, until I finally slipped into depression. Unfortunately, beer and cigarettes became my therapy and escape from reality.

The comradery experienced in basic training was nowhere to be found. I did my best to keep up with the physical training but struggled with the duties of a soldier, like ironing my uniform and shaving in the field. Along with these problems, I began to isolate myself, and my fellow soldiers wanted nothing to do with me, not even giving me a ride to our workplace. I really wanted to be a good soldier, but no one was willing to help me. They made it I was not welcomed.

Finally, one day the other soldiers showed their disdain for me by subjecting me to a hazing incident where I was duct taped into a fetal position. This is when my mental health symptoms emerged in full force. My world split into delusional reality and the real world that other soldiers lived in. These two worlds were constantly competing with each other. Part of my delusional world told me that I had special powers and I thought I could make eye contact and communicate through my brain.

After the hazing, I continued a mental downward spiral. I acted erratically, and I thought I knew what the other soldiers were thinking so I repeated their thoughts aloud which caused them to think I was acting out and being difficult. Now I know that this was the first signs of what would be later diagnosed as schizophrenia. Thinking my “special powers” could help the Army, and I could be a valuable asset, I visited an office with a sign that said, “Mental Health”.

These experiences are in the past, but I often wonder, what would have happened if those in charge had recognized that my erratic behaviors were due to mental health issues. Instead of reducing my pay and assigning extra duties, I wish they had immediately recognized I was not just acting out, but I was struggling with my mental health. Those with authority should be trained to recognize when a soldier is isolated and experiencing mental symptoms. Those soldiers in the Mojave Desert did not see the real me. They saw a soldier who was insubordinate and acting out in numerous ways. They wanted to punish me rather than help me. I did not fit the model of a good soldier, and they did not understand why I could not learn how to be compliant. I, on the other hand, was in turmoil. I desperately needed help, but no one around me knew how to recognize a person who was in mental health crisis.

Recognizing my symptoms and providing help or support would have been a better alternative. When I was in the desert, I thought everyone was against me. After dealing with my shenanigans, those in charge wanted to make sure I was punished. Sometimes people cannot manage life and deserve a mental health refuge instead of punishment. Doing so could save the person’s life. No one gave me that option. Thankfully, I referred myself and began a journey to recovery.

I sincerely hope that my experience is the exception, and not a common occurrence in the military today. Writing this account has been very enlightening for me. I have learned much about mental illness and forgiveness. However, everyone, no matter what their position in life may be, should take it upon themselves to learn about the signs and symptoms of mental illness. After all, one in four of us will be affected by mental illness.

